# Enhancing Hospital Nutrition Assessment Through Artificial Intelligence: A Prospective Tray-Level Pilot Study

**DOI:** 10.3390/nu18081234

**Published:** 2026-04-14

**Authors:** Sofia Favaretto, Honoria Ocagli, Giorgia Shasivari, Paolo Da Rold, Federica Zobec, Solidea Baldas, Chiara Giarracca, Giuseppe Donnarumma, Giulia Lorenzoni, Corrado Lanera, Alois Saller, Dario Gregori

**Affiliations:** 1 Unit of Biostatistics, Epidemiology and Public Health, Department of Cardiac-Thoracic-Vascular Sciences and Public Health, University of Padova, 35121 Padua, Italy; sofia.favaretto@studenti.unipd.it (S.F.); chiara.giarracca@unipd.it (C.G.); giulia.lorenzoni@unipd.it (G.L.); corrado.lanera@unipd.it (C.L.); dario.gregori@unipd.it (D.G.); 2 Department of Internal Medicine, S. Antonio Hospital, 35127 Padua, Italy; alois.saller@aopd.veneto.it; 3 Zeta Research Ltd., 34129 Trieste, Italyfedericazobec@zetaresearch.com (F.Z.); solideabaldas@zetaresearch.com (S.B.); giuseppedonnarumma@zetaresearch.com (G.D.); 4 BIOSTAT-X Biostatistics & AI for Biomedical Discovery, Pediatric Research Institute (IRP) “Città della Speranza”, 35127 Padua, Italy

**Keywords:** disease-related malnutrition, artificial intelligence, image-based dietary assessment, food intake estimation, hospitalized patients, food waste

## Abstract

**Background/Objectives**: Disease-related malnutrition affects 30–50% of hospitalized patients and is associated with adverse outcomes and increased healthcare costs. Routine monitoring of dietary intake typically relies on nursing dietary diaries, which are limited by subjectivity and workload constraints. Artificial intelligence (AI) may improve the accuracy and efficiency of nutritional assessment. This exploratory pilot study evaluated the feasibility of an AI-based system for estimating food intake in hospitalized adults by comparing its performance with gold-standard meal weighing and nurse-completed diaries. **Methods**: A prospective observational study was conducted in the General Medicine Unit of St. Antonio Hospital (Padua, Italy) between June and August 2025. Food intake was assessed using three methods: manual weighing (reference), nursing dietary diaries, and AI-based image analysis. Analyses were performed at the tray level. **Results**: A total of 362 meals from 67 patients were analyzed. Concordance between weighed intake and nursing diaries was 60.8%, with diaries frequently overestimating consumption. In a real-world subset, the AI system achieved a mean absolute error of approximately 40 g (≈10% of average tray weight). As multiple trays could originate from the same patient, uncertainty estimates may be optimistic and should be interpreted with caution. Overall food waste was 30.7% of food served. **Conclusions**: This pilot study shows the feasibility of AI-based intake monitoring in a real-world hospital setting. Our findings are exploratory and based on tray-level analyses; a systematic underestimation bias was observed, and superiority of the AI system over routine documentation cannot be established.

## 1. Introduction

Disease-related malnutrition is a persistent and highly prevalent issue in acute and chronic care, affecting an estimated 20–50% of hospitalized adults [1]. Hospitalization itself often contributes to reduced dietary intake [2], increasing the likelihood of developing or worsening malnutrition during the hospital stay [3,4]. Malnutrition is consistently associated with adverse outcomes, including increased morbidity and mortality, prolonged hospitalization, impaired wound healing, infections, and higher healthcare costs [3]. Evidence shows that malnourished patients may remain in hospital up to 1.5 times longer, incur 24% higher costs, and experience a substantially increased risk of readmission and long-term mortality compared with well-nourished individuals [5]. Accurate assessment of dietary intake is therefore essential, as insufficient intake is a key determinant of malnutrition risk and one of the five diagnostic criteria proposed by the Global Leadership Initiative on Malnutrition (GLIM) [6].

Reduced food intake is typical in hospital settings: more than two-thirds of patients report decreased consumption during admission [2], and approximately 30% of those well nourished at entry become malnourished before discharge; conversely, most patients already malnourished at admission do not improve [7]. Despite the clinical significance of these trends, nutrition is often given limited priority in daily practice. Barriers such as insufficient time, high workloads, inadequate staff training, reduced patient appetite, and limited awareness of nutritional risk contribute to poor and inconsistent monitoring [1]. As malnutrition is multifactorial and dynamic, screening at admission alone is insufficient to detect clinical deterioration, especially among older adults or in the context of shorter hospital stays [7].

Current screening tools, such as the Malnutrition Universal Screening Tool (MUST), provide valuable initial assessments but offer only a static snapshot of nutritional status. They cannot capture daily fluctuations in dietary intake, often one of the first indicators of nutritional decline, and their performance varies between clinical settings [8]. For this reason, international guidelines emphasize the need for structured and regular monitoring. According to ESPEN Recommendation 55, food intake should be assessed at admission, at least weekly in patients not at risk, and daily in those who are malnourished or at risk [9]. Routine monitoring is therefore fundamental for timely nutritional interventions and improved clinical outcomes.

Nurses play a central role in nutritional care due to their continuous interaction with patients and responsibility for documenting intake [10]. However, clinical practice is often constrained by time pressure, competing priorities, and limited training on nutritional evaluation. As a result, documentation of food and fluid intake is often vague, incomplete, or inaccurate. Several studies show that nurses tend to overestimate intake, particularly in patients who eat slowly, require assistance, or consume meals irregularly [10]. Although improving training, promoting individualized assessment strategies, and strengthening interdisciplinary collaboration may enhance the quality of nutritional care [8,10], persistent time and resource limitations highlight the need for supportive technological solutions.

Emerging technologies, particularly artificial intelligence (AI) and machine learning (ML), offer promising opportunities to improve diet monitoring. Traditional methods such as dietary recalls, food frequency questionnaires, or manual records are labour-intensive, subject to recall bias, and limited by inconsistencies in food composition databases [11]. AI-based systems, especially those that rely on image analysis, can support automated identification of food types, estimation of portion sizes, and calculation of energy and macronutrient intake from photographs of meals before and after consumption. Comparative studies suggest that AI-based methods can outperform staff-recorded intake and approach the accuracy of gold-standard weighing, with substantially lower mean errors [12,13,14]. Recent hospital-based studies provide encouraging evidence. A pilot study conducted in France demonstrated the feasibility of automatic image recognition to assess real-life food consumption in hospitalized patients, while also highlighting challenges related to meal heterogeneity and workflow integration [15]. Similarly, an original study from Japan that evaluated more than 300 servings of liquid foods and fruits showed that AI-based estimation was significantly more accurate than visual assessment by staff, particularly for leftovers, underscoring the limitations of subjective estimation [16]. In addition, a scoping review reported that image-based dietary assessment can be effectively applied in hospital settings, particularly among patients with diabetes or dementia [17]. Additional research indicates that AI systems can accurately classify leftover food and detect under-consumption using convolutional neural networks [18]. More recently, a systematic review focusing specifically on hospitalized adults identified 12 quantitative studies evaluating AI-based interventions for the identification or management of malnutrition, concluding that AI tools consistently outperform routine clinical assessment but remain insufficiently integrated into standard care pathways [19].

However, several critical challenges remain, particularly in the segmentation and quantification of heterogeneous meals commonly served in hospital settings. AI systems tend to perform more accurately with visually homogeneous food items, such as meat or fish, which have more consistent shapes and textures, compared to vegetables, whose visual appearance is highly variable [15]. Moreover, liquid evaluation represents an additional challenge for AI-based dietary evaluation, due to their transparency, variable containers, and portion sizes, as highlighted in previous studies [16]. These limitations are especially relevant for texture-modified diets, frequently prescribed to older or dysphagic patients, which are associated with an increased risk of malnutrition [20].

Taken together, the existing evidence highlights a critical gap: while routine monitoring of food intake is essential, current methods remain time-consuming, inconsistent, and prone to human error. AI-based approaches offer a potential solution but require validation in real clinical settings characterized by variability in lighting, food presentation, dishware, and workflow constraints. It is necessary to improve the integration of these systems into daily practice, as well as their ability to provide standardized and objective data, which can enhance the early identification of inappropriate intake and reduce documentation bias.

This study evaluates the feasibility, accuracy, and clinical utility of an AI-based system designed to estimate food intake in hospitalized adults using multi-view image analysis. By comparing AI-generated estimates with gold-standard manual weighing and routine nursing dietary diaries, this pilot study aims to determine whether AI can improve the precision, consistency, and timeliness of nutritional documentation.

## 2. Materials and Methods

### 2.1. Study Design and Participants

An observational design was chosen to evaluate AI-based intake estimation under real-world clinical conditions, without altering routine care or patient behavior. This prospective observational study was conducted in the General Medicine Unit of St. Antonio Hospital (Padua, Italy) between 17 June and 31 August 2025. Although the unit of analysis was the individual meal tray rather than the patient, informed consent was obtained from all participants because study procedures could affect routine care, including the timing of meal delivery compared with other patients. Participants were consecutively recruited from patients admitted to the General Medicine Unit of St. Antonio Hospital during the study period. No a priori sample size calculation was performed, as this was an exploratory validation study conducted under real-world clinical conditions. On each weekday, trained members of the study team reviewed the ward census in collaboration with clinical staff to identify potentially eligible patients. Potentially eligible patients were approached in person, provided with verbal and written information about the study, and invited to participate on a voluntary basis. Written informed consent was obtained prior to any data collection. Financial or other incentives were not offered for participation. Patients who declined to participate or did not meet the eligibility criteria were not included and no data were collected from them.

Eligible patients were ≥18 years old, admitted to the General Medicine Unit, able to select and consume meals independently, and capable of providing informed consent. Exclusion criteria were liquid-only diets (e.g., broth, tea), isolation precautions (contact, droplet, or airborne), cognitive impairment affecting meal intake, and inability to eat autonomously. Only lunch and dinner trays were included because breakfast composition was not standardized.

### 2.2. Outcomes

The primary outcome was the accuracy of AI-based intake estimation compared with manual weighing. Secondary outcomes were the agreement with nursing dietary diaries, AI segmentation performance, and quantification of food waste.

### 2.3. Image Acquisition System (BolC)

**Figure 1:**
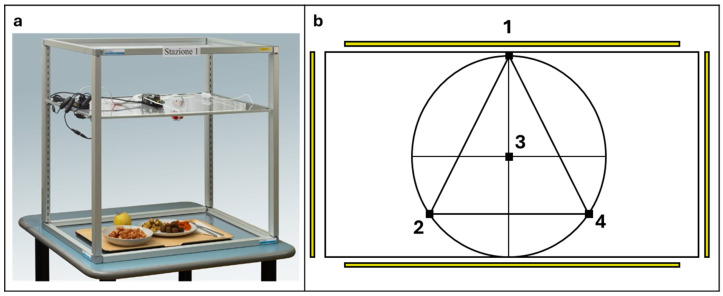
BolC^®^ imaging system and camera configuration. (**a**) Real-world image of the custom-built BolC^®^ acquisition system used in the hospital ward. (**b**) Schematic top-view representation of the camera layout and field of view, illustrating the four-camera configuration used for multi-angle image acquisition.

Meal images were acquired using BolC^®^, a custom-built imaging station developed for this study and shown in Figure 1. The structure consisted of anodized aluminum profiles (25 × 25 mm) supporting three perforated polypropylene panels (60 × 50 cm), which were heat-resistant, washable, and suitable for food contact. Four high-resolution Arducam cameras (4608 × 2592 pixels) were connected to a Raspberry Pi microcomputer. One camera was positioned vertically at 90° for top-down images, while three lateral cameras were mounted at 70° to improve layer detection and volume estimation. The intermediate panel supported both cameras and LED illumination, and the upper panel served as a protective cover. All images were automatically anonymized and assigned a unique identifier linked to the corresponding patient and meal record.

### 2.4. Data Collection

**Figure 2:**
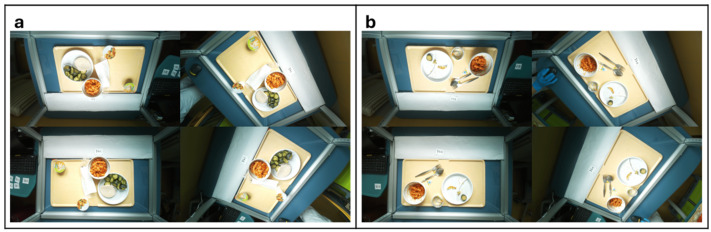
Examples of tray images before (**a**) and after (**b**) consumption.

Data were collected using a standardized workflow. For each enrolled patient, all eligible lunch and dinner trays were consecutively recorded during the study period. Each tray was assigned a unique identifier linking pre- and post-meal images, manual weighing, and nursing dietary diary records. Before the meal, the tray was placed on the BolC^®^ platform, a pre-meal image was captured, and each food item was weighed individually using a calibrated digital scale. After consumption, with no time restrictions for meal duration, a second image was taken, and all remaining components were reweighed. Each tray was assigned a unique alphanumeric identifier to ensure full traceability between images, weights, and clinical records. Representative pre- and post-meal images are shown in Figure 2a,b.

Nursing intake documentation was completed independently as part of routine care, without access to weighing or AI-based estimates. The standard clinical dietary diary was completed using a three-level scale (0 = none, 0.5 = half, 1 = all). All data were digitized and checked for completeness and internal consistency prior to analysis.

All data were subsequently digitized and entered into a structured database for analysis.

### 2.5. Meal Characteristics

Meals were prepared under the supervision of the hospital’s dietary service and typically included a first course (pasta, rice, or soup), a main course with vegetables, and a dessert (fruit, yogurt, or fruit purée). Optional items such as bread or crackers were also recorded. Three representative meal compositions are shown in Table 1.

Meals were provided in three consistent variations (regular, soft-homogeneous, and mashed) and were labeled with specific diet codes. All food items were served in standardized containers, as shown in Figure S1a–c: a bowl for first courses, a flat plate for main dishes and vegetables, and a small bowl for desserts. The dimensions and tare weights of each container were recorded and used as reference parameters for volume and nutrient estimations. Net weight was used for all packaged items.

A structured Excel dataset was created to record all pre- and post-meal weights, meal identifiers, dietary diary values, and image filenames.

Nursing intake estimation is routinely recorded in clinical practice using ordinal categories (none, half, all), rather than as a continuous variable.

### 2.6. AI Segmentation and Volume Estimation

Food components were automatically identified in tray images using an instance segmentation architecture from the Mask R-CNN family (ResNet-50 backbone with Feature Pyramid Network), trained to detect and segment individual food items at pixel level. The training dataset consisted of manually annotated tray images, where food items were labeled with polygonal masks and class identifiers according to a predefined food taxonomy (one label per dish type). Images were acquired from up to four fixed camera views; geometric calibration using reference solids (cubes and cylinders) was performed to estimate per-view spatial scaling factors. Segmentation performance was evaluated independently for each view using accuracy and F1-score, enabling assessment of cross-view consistency.

For portion estimation, segmented mask areas were converted into mass estimates using a learned area-to-weight regression model, operating in single-view or multi-view mode. Training followed a sequential strategy: (i) Mask R-CNN was fine-tuned for food detection and segmentation, then frozen; (ii) the regression head and, when applicable, a confidence-weighted multi-view fusion module were optimized using Smooth L1 loss. To improve generalization with limited real data, the regression stage was pre-trained on synthetically generated mask–weight pairs derived from class- and view-specific statistics of the real dataset. Data were split into training, validation, and test sets at the meal level, with pre- and post-meal images from the same tray kept together to prevent within-tray information leakage. Patient-level splitting could not be implemented because patient identifiers were not available in the analytical dataset, although some patients contributed more than one tray. Accordingly, model evaluation should be interpreted as tray-level validation, and performance uncertainty may be underestimated if observations from the same patient were correlated. AI-based mass estimates were validated against manual weighing, reporting mean absolute error (MAE, grams) and mean absolute percentage error (MAPE).

### 2.7. Nutrient Calculation

Energy and macronutrient intake (protein, carbohydrates, and fat) were calculated using the hospital’s standardized food composition database. Net weight was used for all packaged items.

The unit of analysis was the individual meal tray. For each tray, the following variables were analyzed: pre- and post-meal food weight (grams), consumed food amount (grams), AI-estimated intake (grams), nursing dietary diary score (0, 0.5, 1), diet type, and meal texture category. Agreement between AI-based estimates, manual weighing, and nursing documentation was assessed using concordance measures and error-based metrics, including mean absolute error (MAE).

### 2.8. Statistical Analysis

Descriptive statistical analyses were performed using Jamovi (version 2.6.26), while preliminary data management and consistency checks were conducted in Microsoft Excel.

To enable comparison with routine clinical documentation, continuous intake values derived from manual weighing and AI estimates were mapped to diary categories using predefined thresholds (0–0.2 = none, >0.2–0.7 = half, >0.7–1 = all). The categorization thresholds were derived from the three-level nursing documentation scale (0, 0.5, 1). Agreement and estimation accuracy were evaluated using concordance measures and MAE (grams). Agreement between diary categories and categorized measured intake was evaluated using percent agreement and Cohen’s kappa. Given the ordinal nature of the three intake categories (0, 0.5, 1), the Cohen weighted kappa was used as the primary agreement metric. Kappa estimates were reported with 95% confidence intervals calculated using the standard error. Analyses were conducted in R (R Core Team). A sensitivity analysis was performed to assess the robustness of agreement results to discretization. Nursing intake was categorized into three fixed levels (0%, 50%, and 100%), corresponding to no, half, and full intake, and both AI-based estimates and weighed intake were converted to the same categories. Agreement metrics were recalculated and compared with those obtained from the primary analysis based on continuous intake values.

Agreement between AI-based intake estimates and manual weighing was assessed on the continuous scale using mean signed error (bias), standard deviation of signed errors (precision), and root mean squared error (RMSE). In addition, the proportions of estimates within ±10%, ±15%, and ±30% of the gold standard were calculated (P10, P15, P30). Bland–Altman analysis was performed to estimate mean bias and 95% limits of agreement. Non-parametric bootstrap resampling (5000 iterations) was used to compute 95% confidence intervals for bias, RMSE, and accuracy indices. Because multiple meal trays could be contributed by the same patient, tray-level observations may not be statistically independent. Patient identifiers were not available in the analytical dataset; therefore, patient-level cluster bootstrap, mixed-effects modelling, and cluster-robust variance estimation could not be applied. Consequently, the reported confidence intervals should be interpreted cautiously, as they may be overly narrow. Continuous AI agreement analysis was restricted to trays for which class compatibility and complete weight correspondence were available in the real-world subset.

### 2.9. Use of Artificial Intelligence Tools

No generative artificial intelligence was used for data collection, model development, statistical analysis, or interpretation. Generative AI tools were employed exclusively for non-scientific text editing (grammar and clarity), which does not require formal disclosure under MDPI guidelines.

## 3. Results

A total of 67 patients (21 women, 46 men; age range 47–98 years) were included in the study. The mean age was 78.6 years for women and 74.2 years for men. Across the study period, 362 meals were collected and analyzed. The Physiological Common diet was the most frequent (123; 34.0%), followed by Controlled Carbohydrates 1850 kcal (77; 21.3%) and Controlled Carbohydrates 1500 kcal (52; 14.4%).

### 3.1. Agreement Between Gold-Standard Weighing and Nursing Dietary Diaries

Manual weighing enabled precise calculation of food intake after correction for dishware tare weight. An example of a single-tray analysis is provided in Table 2. Across all trays, the concordance between weighed intake and nursing diary scores was 60.8%. Accuracy varied according to the recorded diary category.

#### 3.1.1. Concordance by Diary Category

The weighted Cohen’s kappa (quadratic weights) indicated moderate agreement between diary and measured intake (κw = 0.49, 95% CI 0.42–0.57), whereas the unweighted kappa suggested fair agreement (κ = 0.31, 95% CI 0.24–0.39).

**Figure 3:**
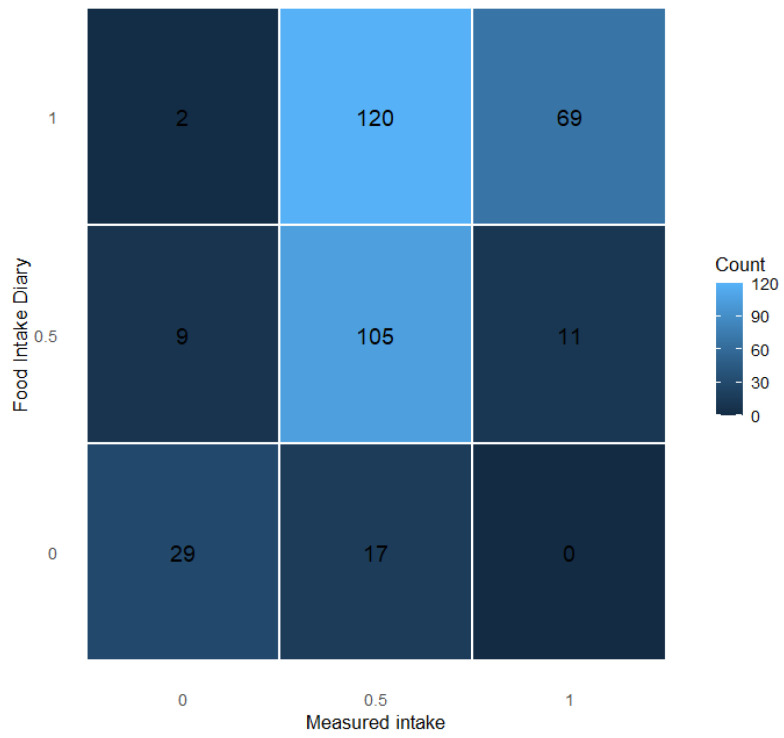
Concordance between food intake diary and measured intake.

When comparing food intake diary records with measured intake, the highest level of agreement was observed for the intermediate category (0.5), with most observations lying on the main diagonal of the concordance matrix. In contrast, records indicating full intake (1) frequently corresponded to partial measured intake, suggesting a tendency toward overestimation at the highest diary level. Misclassification mainly occurred between adjacent categories, while extreme discrepancies were rare. Overall, the intermediate diary category appeared to be the most reliable in reflecting measured intake (Figure 3).

Additional descriptive distributions of measured intake across diary categories are reported in the Supplementary Material (Figure S2). The figure shows the distribution of measured intake across food intake diary categories. The stacked bar plot illustrates the total number of observations for each diary level, with colors indicating the corresponding measured intake categories. A sensitivity analysis treating the nursing intake score as a continuous variable on a 0/50/100 scale yielded consistent results and did not alter the main conclusions (Supplementary Materials—Sensitivity analysis).

#### 3.1.2. Continuous Agreement: Nursing vs. Weighing

For nursing diary estimates (n = 344 trays with non-zero intake), intake scores (0, 0.5, 1) were converted to grams by multiplying the recorded proportion by the pre-meal served weight. The mean signed error was +12.1 g (95% CI −4.7 to 28.3), indicating minimal systematic bias at the population level. However, variability was substantial (SD 159.5 g; RMSE 159.7 g, 95% CI 138.8 to 182.3). Bland–Altman limits of agreement ranged from −300.5 g to 324.7 g. The proportions of estimates within ±10%, ±15%, and ±30% of the gold standard were 28.2% (95% CI 23.5% to 33.1%), 39.8% (95% CI 34.6% to 45.1%), and 65.1% (95% CI 60.2% to 70.1%), respectively (Table 3).

#### 3.1.3. Agreement Analysis Between AI and Gold-Standard Weighing

In the real-world validation subset (n = 23 paired observations), AI-based intake estimates showed a systematic underestimation compared with manual weighing, with a mean signed error (bias) of −41.1 g (95% CI −53.8 to −29.5). The SD of signed errors was 30.9 g and the RMSE was 51.0 g (95% CI 35.7 to 65.1). Bland–Altman analysis yielded 95% limits of agreement from −101.7 g to 19.5 g. The proportions of estimates within ±10%, ±15%, and ±30% of the gold standard were 8.7%, 13.0%, and 30.4%, respectively (Table 3). Given the limited size of this real-world subset, confidence intervals for AI-based agreement metrics were reported only for selected parameters and should be interpreted as exploratory.

### 3.2. AI-Based Food Segmentation and Volume Estimation

The AI system processed paired pre- and post-meal images to perform semantic segmentation and multi-view volume estimation. The corresponding results are reported below.

#### 3.2.1. Food Segmentation Network

Segmentation performances varied according to food type and camera views (Figure S3).

Segmentation accuracy increased during the early training epochs and then stabilized. Foods with well-defined contours (e.g., bread and packaged foods) were segmented with higher accuracy, whereas foods with blended textures, such as mashed vegetables, purées, and soups, showed lower accuracy and reduced F1-scores (Figure S4). Greater heterogeneity in meal composition was associated with poorer segmentation performance.

#### 3.2.2. Volume and Macronutrient Estimation

Building on the segmentation outputs, the AI system estimated consumed volume and the corresponding macronutrient intake. The regression analysis comparing estimated and measured volumes is presented in Figure S5. The mean absolute error (MAE) was approximately 40 g, corresponding to about 10% of the average tray weight. AI-based predictions appeared closer to gold-standard weighing than nursing diary scores in this subset. In contrast, discrepancies in nursing documentation frequently exceeded 100 g, particularly when meals were recorded as fully consumed despite only partial intake.

### 3.3. Food Waste

Food waste was quantified both in absolute terms and relative to the number of trays served.

#### 3.3.1. Overall Waste

**Figure 4:**
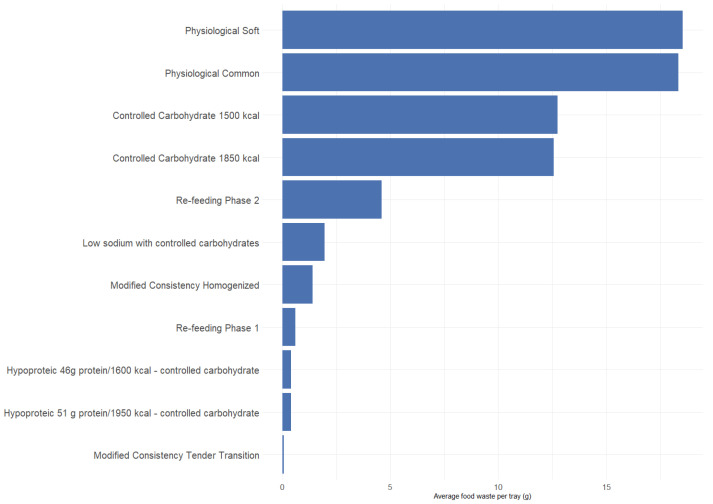
Average food waste per tray by diet type.

Across the 362 trays analyzed, total food waste amounted to 72.1 kg, corresponding to 30.7% of all food served. The distribution of waste across diet categories is presented in Figure 4. The Physiological Soft diet contributed 25.9% of total waste, the Physiological Common diet 25.6%, and the Controlled Carbohydrates 1500 kcal diet 17.8%. Together, these three diets accounted for nearly 70% of total waste.

#### 3.3.2. Waste Adjusted for Meal Frequency

When normalized by the number of trays served, therapeutic diets generated proportionally higher waste. The Low Sodium + Controlled Carbohydrates diet showed the highest waste per tray (62.4% discarded), followed by the Re-Feeding Phase 2 diet (45.0%) and the Physiological Soft diet (42.1%). Thus, while common diets contributed most to the absolute amount of waste due to their greater frequency, therapeutic diets exhibited the highest waste intensity per meal.

## 4. Discussion

In this prospective pilot study, agreement between routine nursing dietary diaries and gold-standard weighing was moderate, with substantial variability across trays. In a real-world validation subset, the AI-based system produced tray-level error estimates characterized by systematic underestimation and measurable variability. These observations should be interpreted as preliminary and exploratory at the tray level. Because repeated trays could not be linked at the patient level, uncertainty estimates and comparative statements should be interpreted cautiously. Overall, these findings should be viewed as preliminary feasibility evidence for AI-assisted intake monitoring rather than definitive validation of superior performance.

Agreement between weighed intake and nursing documentation was limited, with a concordance of only 60.8% and moderate weighted kappa values. This finding aligns with previous research showing the low reliability of subjective intake estimations and the difficulties nurses encounter when documenting nutritional information under demanding workloads [10]. In particular, meals recorded as “fully consumed” were frequently overestimated, suggesting that coarse ordinal categories may be insufficient to capture meaningful variations in intake. Similar observations have been reported by Cass and Charlton, who identified inadequate monitoring as a key contributor to hospital-acquired malnutrition and poorer patient outcomes [1]. By quantifying the magnitude of these discrepancies in an operational clinical setting, the present study further illustrates the limitations of diary-based methods. However, the current study design does not allow determination of whether alternative approaches provide more accurate estimates.

The AI-based system appeared to show lower tray-level dispersion than routine nursing documentation, although a systematic underestimation bias was observed, in the available subset. This comparison should be interpreted with caution, given the inability to account for within-patient correlation. This pattern may suggest greater measurement consistency at the tray level, but not the absence of systematic error. Because patient-level clustered analyses could not be performed, and patient-level train/test separation was not feasible, comparative uncertainty estimates should be interpreted cautiously. While AI achieved a mean absolute error of approximately 40 g (about 10% of average tray weight), agreement metrics should be interpreted cautiously, given the limited size of the real-world subset and class harmonization constraints. Earlier studies suggest that AI-driven approaches may provide more standardized intake estimation compared with routine documentation and provide more stable performance across heterogeneous meal types [13,14]. Nevertheless, accuracy decreased for texture-modified or blended foods, a known challenge for image-based systems and particularly relevant for frail or cognitively impaired patients [20]. This challenge underscores existing methodological constraints and emphasizes the need for further algorithmic refinements, especially in the segmentation and recognition of amorphous or low-contrast food items [19].

The study also documented substantial food waste, with more than 30% of the food served being discarded. This figure is consistent with prior reports indicating hospital waste rates between 30% and 50% [2,7]. Soft and texture-modified diets showed the highest waste levels, reflecting previous evidence of low sensory appeal and reduced acceptance of modified-texture meals among older adults [4,20]. By analyzing waste both in absolute terms and relative to meal frequency, the study provides additional insight into the interaction between diet type, patient vulnerability, and hospital sustainability. These findings reinforce the need for targeted strategies designed to improve the palatability and monitoring of meals in high-risk patient groups.

### 4.1. Limitations

The main methodological limitation of this study concerns non-independence of observations. Because some patients contributed more than one meal tray, within-patient correlation may have affected uncertainty estimates. As patient identifiers were not available in the analytical dataset, we were unable to perform patient-level clustered analyses, including cluster bootstrap or mixed-effects approaches, or to enforce patient-level train/test separation. Therefore, confidence intervals and other uncertainty measures may be overly narrow, and the findings should be interpreted as exploratory tray-level performance estimates rather than definitive patient-level validation.

The study was conducted in a single hospital unit with a relatively small sample size, limiting generalizability. Although manual weighing is considered the gold standard, it is labor-intensive and may introduce minor variations. The AI system also showed reduced accuracy for texture-modified meals. Technical factors, including lighting conditions, camera resolution, and variability in food presentation, may have influenced segmentation performance. Additionally, image acquisition was performed under controlled conditions, a limitation found in another pilot study [15]; thus, the system’s usability in less standardized environments warrants further evaluation. Moreover, the agreement analysis for AI was performed on a limited real-world subset due to incomplete class harmonization and annotation constraints; therefore, these results should be interpreted as feasibility evidence rather than definitive performance benchmarks.

### 4.2. Potential Implications for Practice

Nurses play a pivotal role in nutritional monitoring, yet documentation is often affected by competing clinical demands, limited time, and insufficient training [1,10]. In this pilot study, tray-level AI estimates appeared less variable than routine documentation; however, patient-level clinical implications cannot be inferred from the present data. If confirmed in larger studies with patient-linked analyses, AI-based tools could support documentation and monitoring workflows by providing more standardized tray-level estimates. The high levels of food waste observed, particularly among patients prescribed soft or texture-modified diets, also highlight the clinical and economic implications of unmet nutritional needs. These findings complement international recommendations emphasizing individualized nutritional care and sustainable approaches to hospital food management [9].

Beyond accuracy, integrating AI-based tools into clinical workflows may support workflow standardization, although their clinical impact remains to be demonstrated. However, successful implementation will require addressing organizational and human factors, including digital literacy, resistance to technological change, and concerns regarding professional autonomy. Structured training and institutional support will be necessary to ensure effective adoption.

Operational challenges were also noted with the current BolC configuration, which required trays to be transported to and from the imaging station, introducing workflow disruptions and additional staff workload. Future iterations should focus on embedding compact imaging units directly within meal delivery processes, for example, integrating cameras into meal carts or distribution stations. Automated pre- and post-meal image capture and integration with electronic health records could further streamline monitoring and support real-time decision-making. These developments may ultimately transform nutritional monitoring from a subjective task into a seamless, data-driven component of patient care.

Future research should expand validation to larger and more diverse hospital populations and should prioritize study designs enabling patient-level linkage and appropriate handling of repeated measures. Refining AI algorithms for texture-modified and blended meals remains an important objective. Incorporating depth sensors or 3D reconstruction may enhance volume estimation. Further studies should also evaluate cost-effectiveness and workflow impact in multicenter settings, explore real-time integration with electronic health records and automated alerts, and investigate patient-centered strategies to reduce waste and improve acceptance of therapeutic diets, particularly among individuals at higher nutritional risk.

## 5. Conclusions

This pilot study demonstrates that the implementation of an AI-based system for estimating food intake is feasible within a real-world hospital setting, both from an organizational and workflow perspective. The findings provide preliminary tray-level evidence suggesting that AI-assisted monitoring may support a more standardized and objective assessment of dietary intake and could potentially reduce the burden associated with qualitative nursing documentation.

However, these results should be interpreted as exploratory. Because repeated trays could not be linked at the patient level, uncertainty estimates and comparative performance statements may be optimistic. In addition, AI agreement results were based on a limited real-world subset. Therefore, the superiority of the AI system over routine clinical documentation cannot be established at this stage and requires confirmation in larger studies with patient-level analyses.

Further research is needed to validate these findings in broader and more heterogeneous populations and to determine whether improved measurement consistency translates into meaningful clinical benefits.

## Figures and Tables

**Table 1:** Sample menu with three different combinations.

Meal	First Course	Second Course	Dessert
A	Pasta with tomato	Chicken and carrot	Cooked Apple
B	Vegetable puree with pasta	Beef meatloaf with tomato sauce and potatoes	Fruit puree
C	Rice	Cod medallions and fennel	Yogurt

**Table 2:** Analysis of data for a tray.

Meal	Weight Before (g)	Weight After (g)	Intake (g)	Energy (kcal)	Fat (g)	Carbohydrate (g)	Protein (g)
Pasta with tomato	256	142	114	199.5	36.92	36.92	4.49
Stracchino	100	0	100	314	27	2.6	14
Carrots	79	53	26	16.64	1.86	0	0
Fruit Puree	100	0	100	51	0.5	11	0.9
Total	535	195	340	581.14	34.84	50.53	19.39

**Table 3:** Agreement metrics for AI-based estimates and nursing diary estimates compared with manual weighing.

Metric	AI vs. Weighing (n = 23)	Nursing vs. Weighing (n = 344)
Bias (g)	−41.1 (95% CI −53.8–−29.5)	+12.1 (95% CI −4.7–28.3)
SD of signed error (g)	30.9	159.5
RMSE (g)	51.0 (95% CI 35.7–65.1)	159.7 (95% CI 138.8–182.3)
Bland–Altman 95% LoA (g)	−101.7–19.5	−300.5–324.7
P10 (%)	8.7	28.2 (95% CI 23.5–33.1)
P15 (%)	13.0	39.8 (95% CI 34.6–45.1)
P30 (%)	30.4	65.1 (95% CI 60.2–70.1)

Abbreviations: AI, Artificial Intelligence; SD, Standard Deviation; RMSE, Root Mean Squared Error; LoA, Limits of Agreement; CI, Confidence Interval; P10, Proportion of estimates within ±10% of the gold standard; P15, Proportion of estimates within ±15% of the gold standard; P30, Proportion of estimates within ±30% of the gold standard.

## Data Availability

The raw data supporting the conclusions of this article will be made available by the authors on request.

## Supplementary Materials

The following supporting information can be downloaded at https://www.mdpi.com/article/10.3390/nu18081234/s1. Figure S1: Dishware used for plating; Figure S2: Distribution of measured intake across food intake diary categories; Figure S3: Segmentation model accuracy across epochs by camera view (four-view configuration); Figure S4: Segmentation model F1-score across epochs by camera view (four-view configuration); Figure S5: Regression mean absolute error across epochs for intake estimation (four-view configuration); Sensitivity analysis: Figure S6: Bias and absolute error by nursing category (weighted by counts).

## Abbreviations

The following abbreviations are used in this manuscript:

AI	Artificial Intelligence
MAE	Mean Absolute Error
ML	Machine Learning
GLIM	Global Leadership Initiative on Malnutrition
ESPEN	European Society for Clinical Nutrition and Metabolism
MUST	Malnutrition Universal Screening Tool
