# Supplementary material for: Enhancing Hospital Nutrition Assessment Through Artificial Intelligence: A Prospective Tray-Level Pilot Study
Source: Nutrients. 2026 Apr 14;18(8):1234. doi: 10.3390/nu18081234 (PMC13118341; doi:10.3390/nu18081234)

Enhancing Hospital Nutrition Assessment through Artificial Intelligence: a prospective cohort Pilot Study

Sofia Favaretto [1,2], Honoria Ocagli * [1], Giorgia Shasivari [3], Paolo Da Rold [3], Federica Zobec [3], Solidea Baldas [3], Chiara Giarracca [1], Giuseppe Donnarumma [3], Giulia Lorenzoni [1], Corrado Lanera [1], Alois Saller [2] and Dario Gregori [1,4]

[1] Unit of Biostatistics, Epidemiology and Public Health, Department of Cardiac-Thoracic-Vascular Sciences and Public Health;
[2] Department of Internal Medicine, S. Antonio Hospital, Padua, Italy;
[3] Zeta Research Ltd, Trieste, Italy;
[4] BIOSTAT-X Biostatistics & AI for Biomedical Discovery, Pediatric Research Institute (IRP) "Città della Speranza", Padova, Italy

Supplementary materials

Figure S1 - Dishware used for plating 2

Figure S2 - Distribution of measured intake across food intake diary categories. 3

Figure S3 - Accuracy per View – 4 View configuration. 4

Figure S4 - F1-score per view – 4 View configuration 5

Figure S5 - Final Regression MAE – 4 View 6

Supplementary Materials

Figures

Figure S1 - Dishware used for plating

(a) bowl for first courses;

(b) flat plate for mains and vegetables;

(c) small bowl for cooked apple.

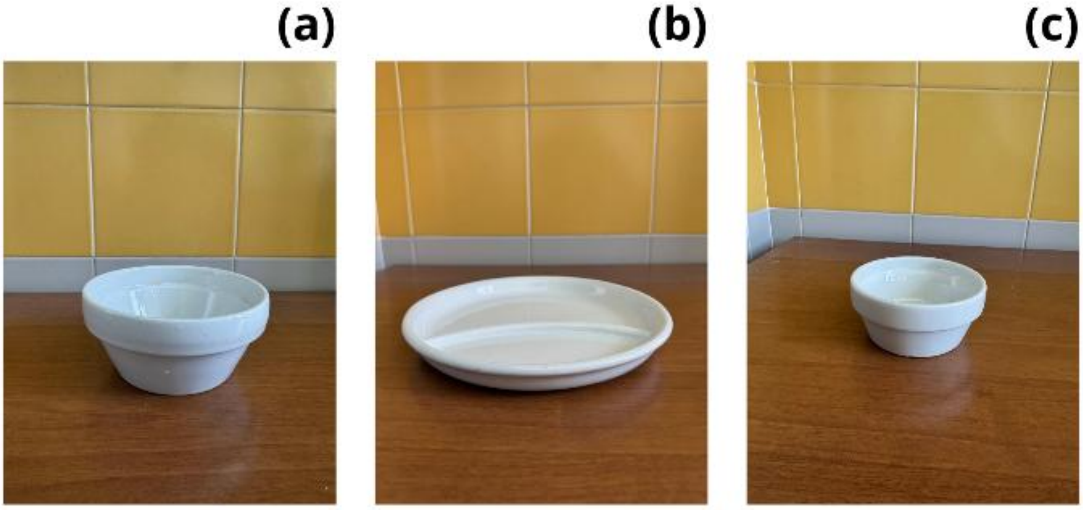

Figure S2 - Distribution of measured intake across food intake diary categories

The stacked bar plot illustrates the total number of observations for each diary level, with colors indicating the corresponding measured intake categories. This figure is intended to provide descriptive context on the distribution of observations and does not represent a formal assessment of concordance.

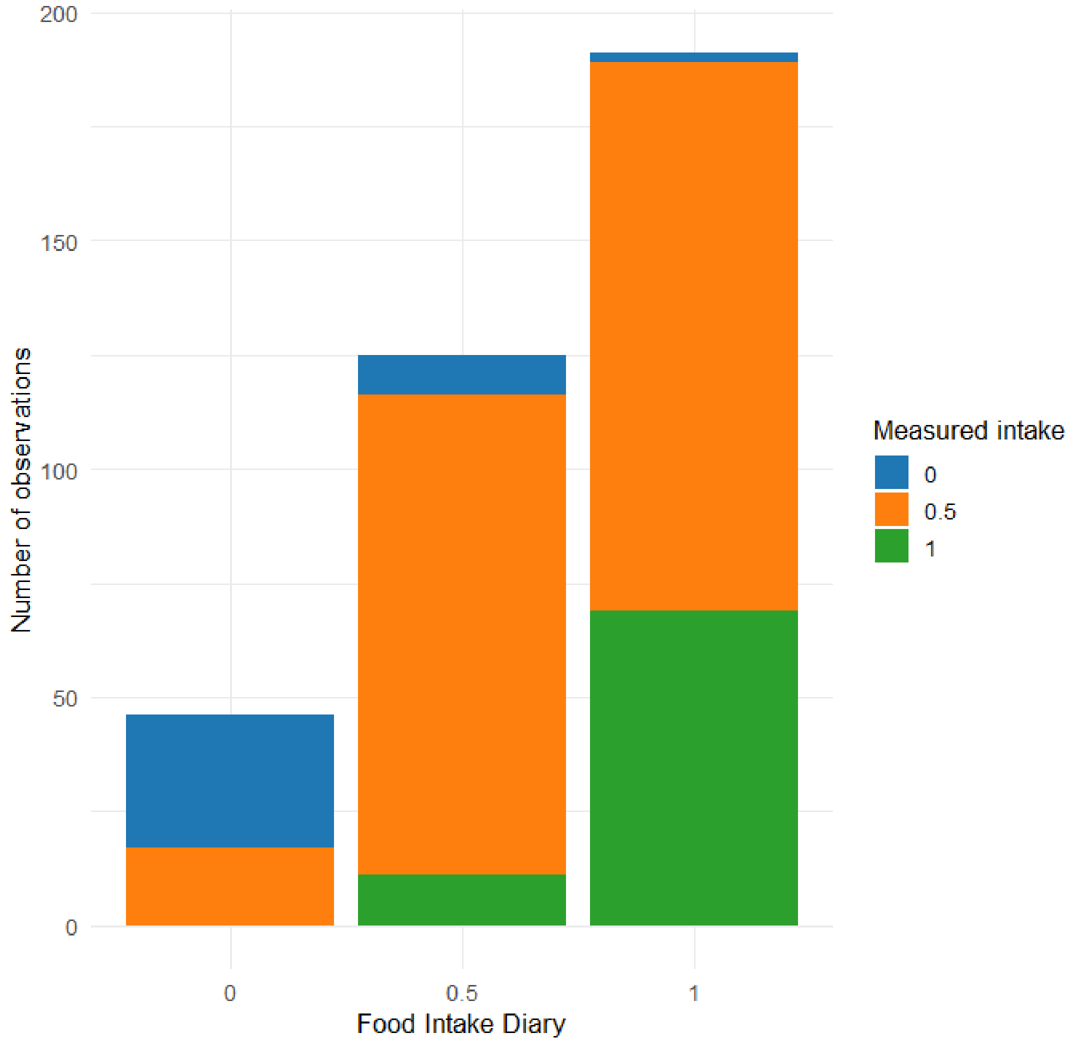

Figure S3 - Segmentation model accuracy across epochs by camera view (four-view configuration)

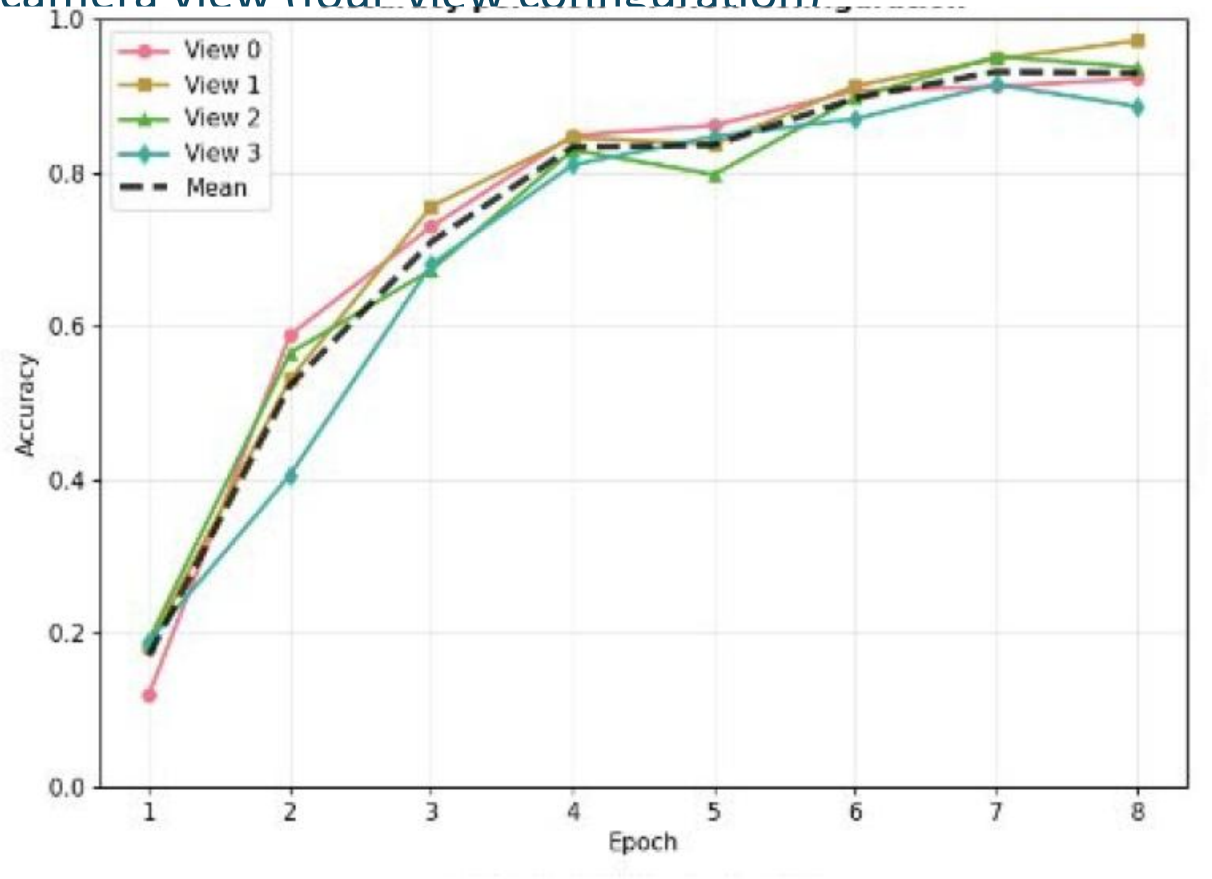

Figure S4 - Segmentation model F1-score across epochs by camera view (four-view configuration)

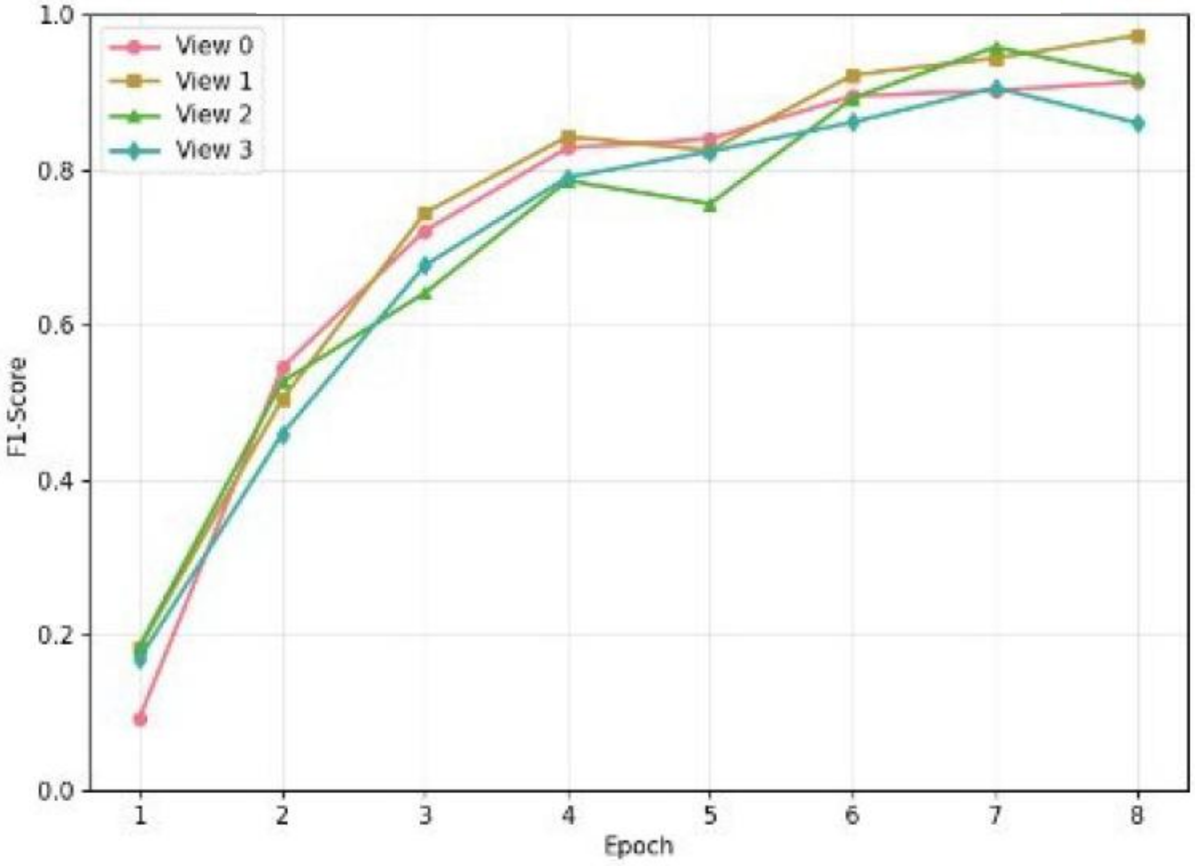

Figure S5 - Regression mean absolute error across epochs for intake estimation (four-view configuration)

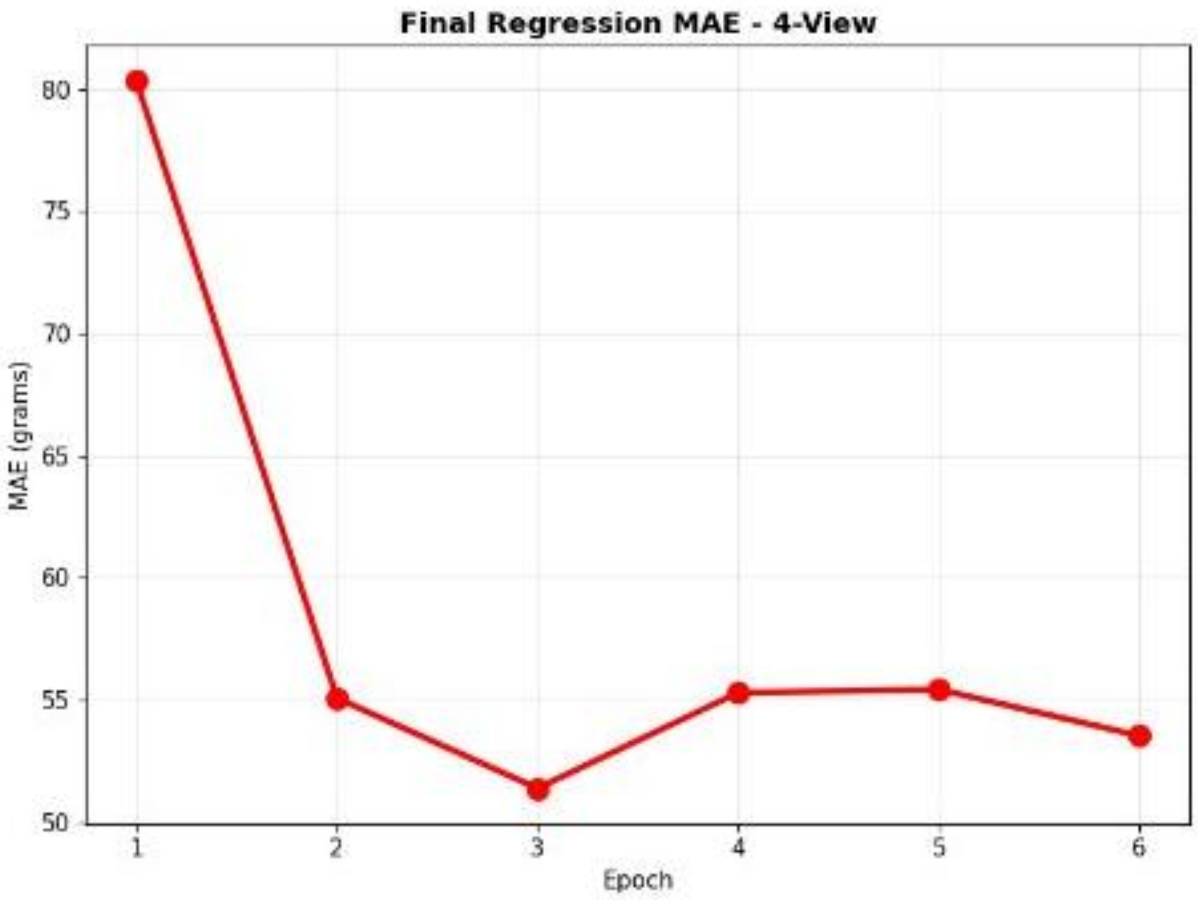

Sensitivity analysis

The nursing intake score (0, 0.5, 1) was recoded to a 0/50/100 scale and compared with measured intake on the same scale. Bias and mean absolute error were calculated by nursing category, weighted by the number of observations. The distribution of differences (measured − nursing) confirmed the main findings: agreement was highest at the intermediate level, while the highest nursing category showed the greatest tendency toward overestimation. Overall conclusions were unchanged.

Figure S6 – Bias and absolute error by nursing category (weighted by counts)

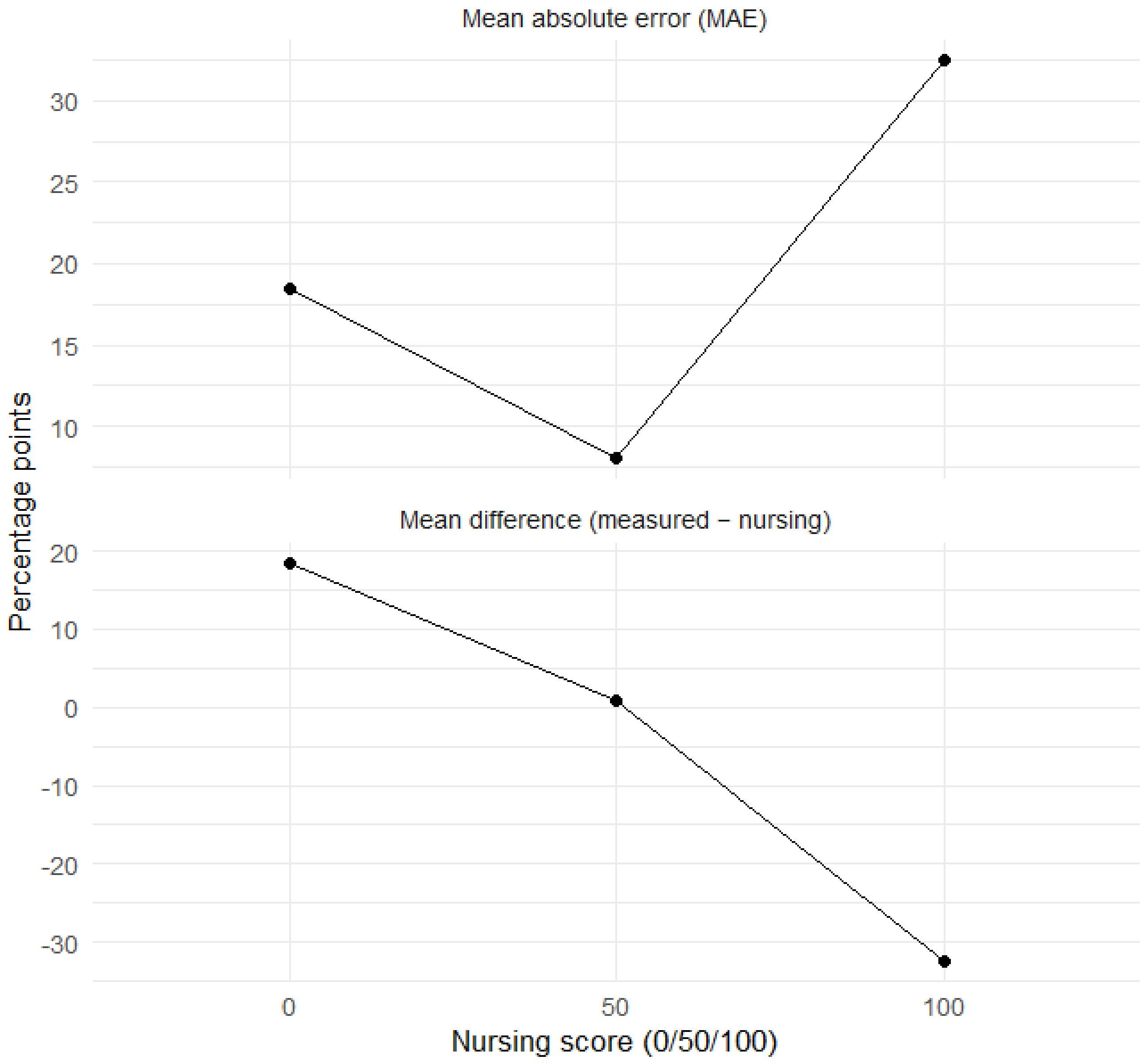

Supplement: Supplementary file 1 [file nutrients-18-01234-s001.zip › nutrients-4095975-supplementary.pdf]
